# The additional value of ^18^F-FDG PET/CT imaging in guiding the treatment strategy of non-tuberculous mycobacterial patients

**DOI:** 10.1186/s12931-024-02757-7

**Published:** 2024-03-18

**Authors:** Donghe Chen, Yunbo Chen, Shuye Yang, Kanfeng Liu, Zhen Wang, Tingting Zhang, Guolin Wang, Kui Zhao, Xinhui Su

**Affiliations:** 1 Department of Nuclear Medicine, The First Affiliated Hospital, Zhejiang University School of Medicine, 79 Qingchun Road, Hangzhou, 310003 China; 2 State Key Laboratory for Diagnosis and Treatment of Infectious Diseases, The First Affiliated Hospital, Zhejiang University School of Medicine, Hangzhou, 310003 China

**Keywords:** Non-tuberculous mycobacteria, NTM-PD, Disseminated NTM infection PET/CT, ^18^F-FDG

## Abstract

**Objectives:**

Non-tuberculous mycobacteria (NTM) infection is an increasing health problem due to delaying an effective treatment. However, there are few data on ^18^F-FDG PET/CT for evaluating the status of NTM patients. The aim of this study was to investigate the potential value of ^18^F-FDG PET/CT in guiding the treatment strategy of NTM patients.

**Methods:**

We retrospectively analyzed the cases of 23 NTM patients who underwent ^18^F-FDG PET/CT. The clinical data, including immune status and severity of NTM pulmonary disease (NTM-PD), were reviewed. The metabolic parameters of ^18^F-FDG included maximum standardized uptake value (SUV_max_), SUV_max_ of the most FDG-avid lesion (SUV_Top_), SUV_Top_/SUV_max_ of the liver (SUR_Liver_), SUV_Top_/SUV_max_ of the blood (SUR_Blood_), metabolic lesion volume (MLV), and total lesion glycolysis (TLG). The optimal cut-off values of these parameters were determined using receiver operating characteristic curves.

**Results:**

There were 6 patients (26.09%) with localized pulmonary diseases and 17 patients (73.91%) with disseminated diseases. The NTM lesions had high or moderate ^18^F-FDG uptake (median SUV_Top_: 8.2 ± 5.7). As for immune status, the median SUV_Top_ in immunocompromised and immunocompetent patients were 5.2 ± 2.5 and 10.0 ± 6.4, respectively, with a significant difference (*P =* 0.038). As for extent of lesion involvement, SUR_Liver_ and SUR_Blood_ in localized pulmonary and disseminated diseases were 1.9 ± 1.1 vs. 3.8 ± 1.6, and 2.7 ± 1.8 vs. 5.5 ± 2.6, respectively, with a significant difference (*P* = 0.016 and 0.026). Moreover, for disease severity, SUV_max_ of the lung lesion (SUV_I−lung_) and SUV_max_ of the marrow (SUV_Marrow_) in the severe group were 7.7 ± 4.3 and 4.4 ± 2.7, respectively, significantly higher than those in the non-severe group (4.4 ± 2.0 and 2.4 ± 0.8, respectively) (*P* = 0.027 and 0.036). The ROC curves showed that SUV_Top_, SUR_Liver_, SUR_Blood_, SUV_I−lung_, and SUV_Marrow_ had a high sensitivity and specificity for the identification of immune status, lesion extent, and severity of disease in NTM patients.

**Conclusion:**

^18^F-FDG PET/CT is a useful tool in the diagnosis, evaluation of disease activity, immune status, and extent of lesion involvement in NTM patients, and can contribute to planning the appropriate treatment for NTM.

**Supplementary Information:**

The online version contains supplementary material available at 10.1186/s12931-024-02757-7.

## Introduction

Non-tuberculous mycobacteria (NTM) are *Mycobacterium* (*M.*) species other than *M. leprae* and *M. tuberculous*. NTM are prevalent in the environment, such as soil, dust, plants, water sources, foods, and animals, and are more likely to infect immunocompromised than immunocompetent individuals [1]. The prevalence of diseases caused by NTM has increased in recent years; for instance, in the United States, the number of people affected rose from 40 to 85 per 100,000 individuals between 2010 and 2014 [2]. In China, NTM-related diseases have also gradually become more common over the last 20 years [3], with more than 170 NTM species reported [4, 5].

NTM can cause a variety of clinical syndromes ranging from lymphadenopathy to aseptic meningitis [6, 7]. The most common clinical manifestation of NTM infection is NTM pulmonary disease (NTM-PD). Furthermore, extra-pulmonary NTM diseases, such as infections of soft tissues, lymph nodes or bone, can emerge as a result of NTM exposure [5]. The primary diagnostic methods are culture of clinical samples, and molecular and genetic detection (including DNA PCR assay, metagenomic next-generation sequencing (mNGS) from body fluid or histopathological tissue) [9]. However, these methods are invasive, requiring biopsies from patients. In addition, the sensitivity and accuracy of these procedures are limited due to sampling errors or contamination, as well as NTM heterogeneity within biopsy samples [8, 9].

Computed tomography (CT) has been widely used in the diagnosis of pulmonary diseases. However, NTM-PD have poor specific radiological manifestations. NTM can also infect organs and tissues besides lungs, such as the skin, bones and soft tissues, also known as disseminated NTM infections due to immune status [5]. The current guidelines for NMT treatment, such as the Official ATS/ERS/JRS/ALAT Clinical Practice Guideline for Treatment of NTM in 2020 [10] and the British Thoracic Society guidelines for the management of NTM pulmonary disease in 2017 [11], strongly recommend that individuals may require a period of longitudinal assessment (including immune status, the severity and risk of progressive NTM infection, and the extent of lesion involvement) before a decision is made to start treatment. Therefore, it is critical to develop a noninvasive imaging tool for evaluating active lesion, immune status, and the extent of lesion involvement [12].

^18^F-FDG (2-[^18^F]-fluoro-2-deoxy-D-glucose) is a non-physiological glucose analogue, which is metabolized by the same physiological processes as glucose [13]. Inflammatory cells with multiple mechanistic similarities to malignant cells, such as macrophages, demonstrate greater FDG uptake [13]. ^18^F-FDG PET/CT has been used for detecting inflammation and infection such as fever of unknown origin (FUO), sarcoidosis, IgG4-related systemic disease, and human immunodeficiency virus (HIV)-related infections, among others. Its roles in diagnosis include mapping extent of disease, assessing therapy response based on whole-body imaging, and changes in FDG metabolic parameters ahead of morphological changes by conventional imaging techniques (CT and MRI) [14–16]. Moreover, ^18^F-FDG PET/CT has been suggested as a potential tool for evaluating the responsiveness of tuberculosis (TB) infection to anti-tuberculosis therapy [17, 18]. A few case reports have indicated a possible value of ^18^F-FDG PET/CT for patients with NTM [19–22]. However, available data concerning the findings of ^18^F-FDG PET/CT in NTM has been limited. Furthermore, according to the guidelines for the management of non-tuberculosis mycobacterial pulmonary disease by the British Thoracic Society [11], the choice of therapeutic strategy relies strongly on the immune status, extent of lesion involvement, and disease severity in NTM patients. Meanwhile, there are limited data on the use of ^18^F-FDG PET/CT for evaluating the status of NTM patients. Therefore, this retrospective study was performed to investigate and confirm the potential value of ^18^F-FDG PET/CT in guiding the treatment strategy in NTM patients.

## Materials and methods

### Patient information

**Fig. 1:**
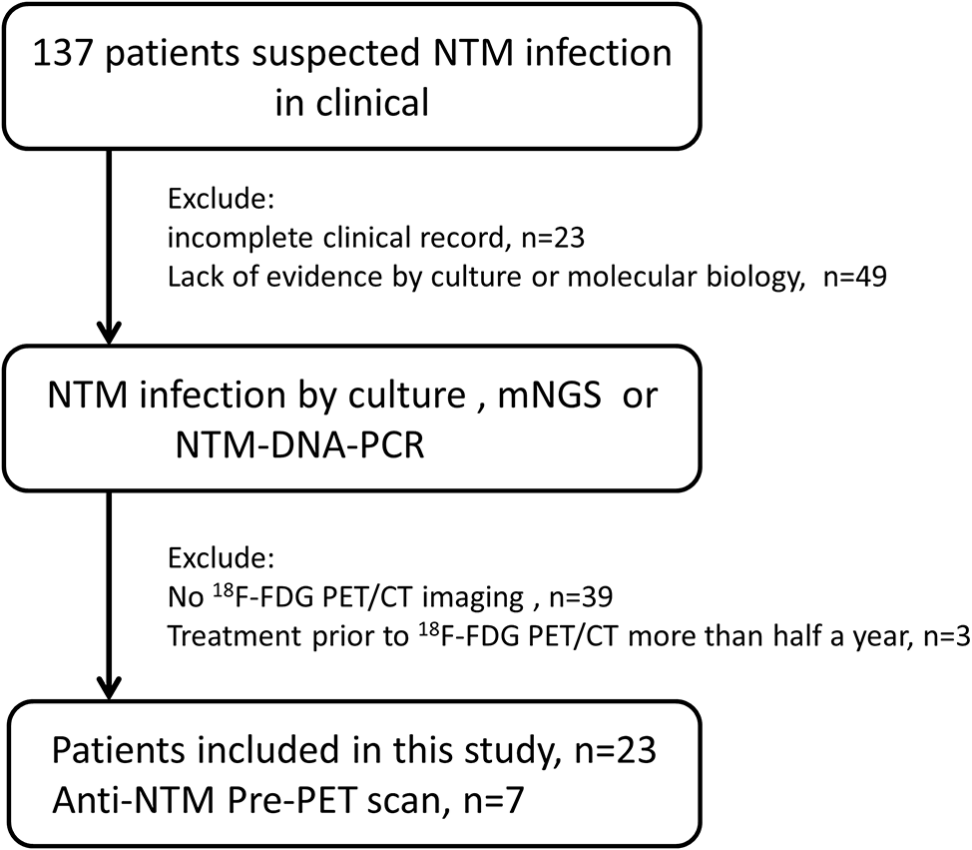
Flow diagram showing the patient selection details. NTM: Non-tuberculous mycobacteria; ^18^F-FDG PET/CT: ^18^F-fluorodeoxyglucose positron emission tomography/computed tomography; mNGS: Metagenomic next-generation sequencing; NTM-DNA-PCR: PCR assay for non-tuberculous mycobacterial DNA

We retrospectively reviewed 23 patients with a confirmed diagnosis of NTM infection who underwent ^18^F-FDG PET/CT between December 2016 and October 2021 in the First Affiliated Hospital, Zhejiang University School of Medicine. The flow diagram of the study design is presented in Fig. 1. The clinical information (age, gender, risk factor, and syndrome) and laboratory test results (serum cytokines, immune cell, and mycobacterium species) were obtained by reviewing the available medical records. A total of 16 patients underwent ^18^F-FDG PET/CT imaging before anti-NTM therapy, while the other 7 patients underwent ^18^F-FDG PET/CT imaging after anti-NTM treatment (within less than 6 months).

Based on the risk factors of immune status, the 23 enrolled patients were divided into two groups: immunocompromised patients and immunocompetent patients. According to a previous article [23], immunocompromised patients were defined as follows: those with primary immune deficiency disease; active malignancy; receiving cancer chemotherapy; HIV infection with a CD4 T-lymphocyte count < 200 cells/mL; solid organ transplantation; hematopoietic stem cell transplantation; receiving corticosteroid therapy with a dose 20 mg prednisone or equivalent daily for ≥ 14 days or a cumulative dose > 700 mg of prednisone; receiving biologic immune modulators (such as azathioprine, corticosteroids and mesalazine etc.); receiving disease-modifying anti-rheumatic or other immunosuppressive drugs. Based on the extent of lesion involvement in NTM patients [24, 25], these 23 patients classified into two groups: localized pulmonary diseases group and disseminated diseases group. The patients with NTM disease involvement only in lungs were defined as localized pulmonary diseases, while the patients with NTM disease involvement both in lungs and other tissues or organs (such as skin, lymph nodes, bone and joint, soft tissue, urinary system, etc.) were defined as disseminated diseases. According to the disease severity, these patients were further classified into severe and non-severe disease groups based on the British Thoracic Society guidelines for the management of NTM-PD in 2017 [5, 11]. Non-severe disease included: (a) mild-moderate symptoms, (b) no signs of systemic illness, (c) absence of lung cavitation and extensive lung disease, (d) acid-fast bacilli (AFB) smear-negative in the pulmonary specimens. Severe disease included: (a) presence of severe symptoms and signs of systemic illness, (b) presence of lung cavitation and extensive lung involvement, (c) pulmonary specimens positive for acid-fast bacilli (AFB) smear.

### ^18^F-FDG PET/CT imaging

^18^F-FDG was synthesized using a Siemens Eclipse cyclotron (Siemens Medical Solutions, Knoxville, TN 37,932, USA) and a Siemens Explora FDG4 chemical synthesis module. The patients were fasted for at least 4 h before being injected with ^18^F-FDG at a dose of 4.44 to 5.55 MBq/kg. Images were acquired using a PET/CT instrument (Biograph 16 and Version, Siemens Company, Germany). Two experienced nuclear medicine physicians independently reviewed PET/CT images until consensus was reached. Semiquantitative analysis was performed using the maximum standardized uptake value (SUV_max_). Furthermore, 10 metabolic parameters were also recorded: SUV_Top_ (SUV_max_ of the most FDG-avid lesion), SUV_I−Lung_ (SUV_max_ of intra-pulmonary lesions), SUV_E−Lung_ (SUV_max_ of extra-pulmonary lesions), SUV_Liver_ (SUV_max_ of the liver), SUV_Spleen_ (SUV_max_ of the spleen), SUV_Marrow_ (SUV_max_ of the marrow), SUR_Liver_ (the most FDG-avid lesion-to-liver SUV_max_ ratio) and SUR_Blood_ (the most FDG-avid lesion-to-blood pool SUV_max_ ratio), metabolic lesion volume (MLV) and total lesion glycolysis (TLG). The MLV of each lesion was calculated using an automated region-growing algorithm with SUV thresholds of 2.5. The TLG was measured as MTV multiplied by the average SUV of each lesion.

### Statistical analysis

Quantitative data were presented as mean and standard deviation. All statistical analyses were performed using GraphPad Prism, version 8.0 (GraphPad Software, San Diego, CA, USA). The differences in immune status, extent of lesion involvement and severity of NTM-PD were analyzed using the chi-squared test. A *p*-value of less than 0.05 was considered statistically significant. ROC curves were used to determine the optimal cut-off values.

## Results

### Patients’ clinical characteristics

The demographics and clinical features of the 23 patients with NTM infection were summarized in Table 1. These patients included 19 (82.6%) men and 4 (17.4%) women, with an age range between 14 and 84 years (median, 60.0 years). The diagnosis of these patients was confirmed by tissue culture (*n* = 6), non-tuberculous mycobacteria DNA PCR assay (NTM-DNA-PCR) (*n* = 3), and metagenomic next-generation sequencing (mNGS) (*n* = 14). Specimens were collected from blood or bronchoalveolar lavage fluid (BALF), pus, sputum, tissue of lung lesions, and bone lesions. The most frequent NTM species detected was *M. intracellulare* (12 cases, 52.1%), and 1 patient had mixed infection with *M. intracellulare* and *M. kansasii*.

**Table 1:** Demographics and clinical characteristics of patients with NTM infection

No.	Gender	Age	Bacterial species	Risk factor	Clinical symptoms	Diagnosis	Involved sites	Anti-NTM Pre-PET scan	SUV_Top_	MLV	TLG	PET/CT findings
1	M	38	*M. abscessus*	COPD	Fever, cough	mNGS (BALF)	Bilateral lungs	None	11.3	161.3	578.8	Pulmonitis
2	M	58	*M. intracellulare*	COPD	Ostealgia	Culture (Pus)	Right lung, multiple bones	None	14.8	438.8	1802.5	MM
3	M	71	*M. intracellulare*	Pulmonary TB history	Fatigue, cough	TB-DNA-PCR (Sputum)	Bilateral lungs, lymph nodes, adrenals	None	11.8	972.6	4628.6	Pulmonitis
4	M	84	*M. intracellulare*	COPD	Cough	Culture (Blood)	Bilateral lungs, lymph nodes	Oral ofl 1weeks	7.5	42.6	148.7	Pulmonitis
5	M	29	*M. abscessus*	AIDS	Fever	Culture (lymph node)	Bilateral lungs, lymph nodes	Oral Azi + Eth + Rif 3months	6.6	48.2	128.4	Lymphadenoma
6	M	79	*M. intracellulare*	COPD	Chest tightness	mNGS (BALF)	Left lung, lymph nodes	None	4.1	27.3	79.7	Pulmonitis
7	M	50	*M. chimaera*	Chemotherapy of lymphoma	None	mNGS (BALF)	Bilateral lungs	None	2.4	0	0	Pulmonitis
8	M	61	*M. intracellulare*	Splenectomy	Ostealgia	mNGS (bone surgery)	Multiple bones, lymph nodes	None	6.0	58	200.3	MM
9	M	44	*M. kansasii*	None	Ostealgia	mNGS (bone biopsy)	Bilateral lungs, lymph nodes, multiple bones, subcutaneous	None	28.1	129.9	637.7	Lung cancer and multiple metastases
10	F	60	*M. abscessus*	Chemotherapy of leukemia	None	TB-DNA-PCR (Sputum)	Bilateral lungs	Oral Azi + Eth + Rif 5 months	1.2	0	0	Pulmonitis
11	F	14	*M. Avium*	MonoMAc syndrome	Fever, cough	mNGS (lung puncture)	Bilateral lungs, lymph nodes	None	5.1	70.9	244.1	Pulmonitis
12	M	63	*M. intracellulare*	History of lung cancer.	Ostealgia	mNGS (bone biopsy)	Bilateral lungs, lymph nodes, multiple bones	None	7.9	137.6	539.1	Bone metastases
13	M	19	*M. Avium*	Hepatitis B cirrhosis	Fever	TB-DNA-PCR (Sputum)	Right lung, lymph nodes	Oral Eth + Rif 1 weeks	5.5	60.3	211.2	Pulmonitis
14	M	60	*M. paraintracellulare*	Chemotherapy of lung cancer	cough	mNGS (BALF)	Bilateral lungs, left 7 rib	Oral Azi + Eth + Rif 2 months	4.5	27.8	88.4	Recurrence of lung cancer
15	F	70	*M. intracellulare*	None	Fever, ostealgia	mNGS (lung puncture)	Left lung, multiple bones	None	13.8	31.7	221.7	Lung cancer and metastases
16	F	60	*M. intracellulare*	Splenectomy	Fever, cough	Culture (Sputum)	Bilateral lungs, lymph nodes	None	13.0	309.8	1283.5	Pulmonitis
17	M	78	*M. intracellulare + M. kansasii*	COPD	Weight loss	mNGS (BALF)	Left lung	Oral Azi + Eth + Rif 2 months	8.3	0.7	2.6	Pulmonitis
18	M	73	*M. Avium*	None	Fever, cough	mNGS (lung puncture)	Bilateral lungs, lymph nodes	None	5.8	44.9	137.9	Lung cancer and metastases
19	M	61	*M. intracellulare*	Chemotherapy of Lung MALToma	Fatigue	mNGS (BALF)	Bilateral lungs, lymph nodes	None	5.2	135.1	666.1	Recurrence of lung maltoma
20	M	27	*M. Avium*	AIDS	Fever	Culture (Blood)	Bilateral lungs, lymph nodes, spleen	Oral Cla + Mox + Eth + Rif 3 months	6.4	21.5	176.2	Pulmonitis
21	M	66	*M. intracellulare*	COPD	Cough	Culture (Sputum)	Bilateral lungs	None	3.4	1.2	3.3	IIP
22	M	61	*M. abscessus*	Chemotherapy of pancreatic cancer	Fever	mNGS (BALF)	Bilateral lungs	None	6.3	38	124.8	Pulmonitis
23	M	55	*M. intracellulare*	Chemotherapy of MDS	Fever	mNGS (BALF)	Bilateral lungs, lymph nodes	None	9.9	52.4	216	Invasion of tumor

COPD: Chronic obstructive pulmonary disease; TB: Tuberculosis; AIDS: Acquired immunodeficiency syndrome; MDS: Myelodysplastic syndromes; mNGS: Metagenomic next-generation sequencing; BALF: Bronchoalveolar lavage fluid; TB-DNA-PCR: Mycobacterium tuberculosis DNA; NTM: Non-tuberculous mycobacterial; Ofl: Ofloxacin; Azi: Azithromycin; Eth: Ethambutol; Rif: Rifampicin; Cla: Clarithromycin; Mox: Moxalactam; MM: multiple myeloma; IIP: idiopathic interstitial pneumonia

In terms of the risk factors of immune status, among the 23 patients, 9 (39.1%) were immunocompromised, including during chemotherapy (6 cases, 26.1%), due to lymphoma (2 cases), leukemia (one case), MDS (one case), lung cancer (one case), pancreatic cancer (one case), acquired immune deficiency syndrome (AIDS) (2 cases, 8.7%), and monocytopenia and mycobacterial infection syndrome (MonoMAc syndrome) (1 case, 4.3%). The other 14 patients were immunocompetent, of which 5 patients (37.8%) presented with underlying diseases, such as splenectomy (2 case, 8.6%), hepatitis B cirrhosis (1 case, 4.3%), tuberculosis (1 case, 4.3%), and early-stage lung cancer (1 case, 4.3%). Six patients exhibited a mild chronic obstructive pulmonary disease (COPD) because of long-term smoking, whereas 3 patients had no risk factors or underlying diseases.

### Laboratory characteristics

The laboratory characteristics of the 23 patients with NTM infection were presented in Supplementary Table 1. The median white cell count was 7.6 × 10^9^/L (range: 2.3–22.8 × 10^9^/L), and 10 patients (43.5%) had leukocytosis (10 cases, 43.5%). The median platelet count was 226 × 10^9^/L (range: 85–492 × 10^9^/L), and 8 patients (34.8%) had thrombocytosis. The majority of patients showed varying levels of increase in the C-reactive protein (CRP) (16 cases, 69.6%), erythrocyte sedimentation rate (ESR) (15 cases, 65.2%) and interleukin 6 (IL-6) level, (20 cases, 87%). The other laboratory test results were almost within the normal range, including procalcitonin (PCT), serum cytokines, and cellular immune function in the peripheral blood.

### ^18^F-FDG PET/CT imaging characteristics

The ^18^F-FDG PET/CT imaging characteristics of the 23 patients with NTM infection were summarized in Table 2.

**Table 2:** imaging characteristic of ^18^F-FDG PET/CT in patients with NTM infection

Imaging characteristic	n (range or %)
**Intra-pulmonary FDG accumulation**	22(95.7)
Single lobe	5(21.7)
Double lobe	17(73.9)
**Morphological manifestations in the lung**	
Patchy infiltration	18(81.8)
Nodular infiltration	3(13.6)
Bronchiectasis	8(36.4)
Cavitation	5(22.7)
Calcification	3(13.6)
Atelectasis	2(9.1)
Pleural effusion	4(18.2)
**Extra- pulmonary FDG accumulation**	17(73.9)
Lymph nodes	14(60.9)
Bone	6(26.1)
Subcutaneous	1(4.3)
Spleen	1(4.3)
Adrenal gland	1(4.3)
**Extent of lesion involvement**	
Pulmonary NTM group	6(26.1)
Disseminated NTM group	17(73.9)
**Metabolic parameters**	mean (range)
SUV_Top_	8.2 ± 5.7 (1.2–28.1)
SUV_I−lung_	6.6 ± 3.9 (1.2–13.9)
SUV_E−lung_	6.3 ± 6.3 (1.8–28.1)
SUV_Liver_	2.7 ± 0.6 (1.7–3.8)
SUV_Marrow_	3.7 ± 2.0 (1.7–6.7)
SUV_Spleen_	2.5 ± 0.9 (1.7-6.0)
SUR_Liver_	3.7 ± 2.3 (0.5–7.6)
SUR_Blood_	5.9 ± 3.9 (0.7–10.3)
MLV (cm^3^)	122.2 ± 211.7 (0-972.6)
TLG(ml*SUV)	526.9 ± 992.1 (0-4628.6)

^18^F-FDG PET/CT: ^18^F-fluorodeoxyglucose positron emission tomography/computed tomography; NTM: Non-tuberculous mycobacterial infection; SUV_Top_: The most FDG-avid lesion in the whole body; SUV_I−lung_: SUV_max_ of lung lesions, SUV_E−lung_: SUV_max_ of extra-lung lesions; SUV_Liver_: SUV_max_ of liver; SUV_Spleen_: SUV_max_ of spleen; SUV_Marrow_: SUV_max_ of marrow; SUR_Liver_: SUV_max_ ratio of lesion-to-liver; SUR_Blood_: SUV_max_ ratio of lesion-to-blood pool. MLV: the metabolic lesion volume; TLG: total lesion glycolysis

#### Intra-pulmonary lesions

**Fig. 2:**
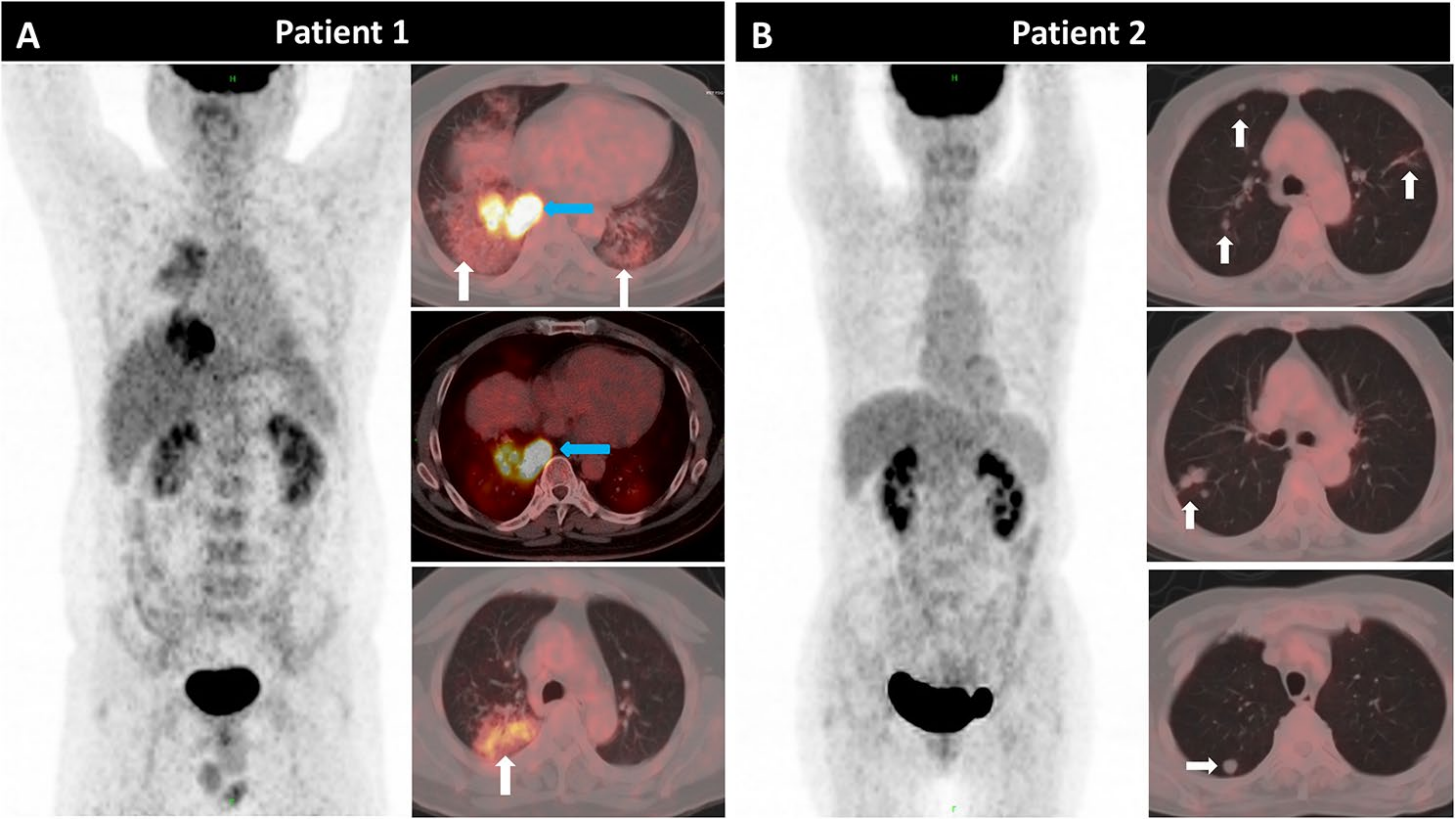
Patient 1 (No.1): A 38-year-old male patient presented with cough, phlegm and intermittent fever for two months. The pathology of the pulmonary puncture suggested chronic granulomatous inflammation of the lungs. Finally, metagenomics next-generation sequencing (mNGS) of alveolar lavage fluid confirmed *M. abscessus* infection. Multiple patchy shadows (white arrows, SUV_max_ = 11.3) and consolidation (blue arrows, SUV_max_ = 3.7) in both lungs on the axial slices of ^18^F-FDG PET/CT scan images.Patient 2 (No.10): A 60-year-old female patient with a history of acute lymphocyte leukemia. After one cycle of chemotherapy, opportunistic intracellular mycobacterial infection appeared in both lungs and was confirmed by TB-DNA-PCR of the sputum. The patient was given active anti-infection treatment (oral azithromycin + ethambutol + rifampicin for 5 months). To assess the tumor activity after treatment, an ^18^F-FDG PET/CT scan was performed, which revealed multiple FDG-unavid nodules (white arrows, SUV_max_ = 2.2) in the right lung. The pathology of the wedge resection of the right lung nodule indicated pulmonary granulomatosis

^18^F-FDG PET/CT revealed NTM-PD in 22 patients (95.6%) with lung involvement, including the involvement of a single lobe (5 cases) and two lobes (17 cases). Among these patients, the common CT feature was the presence of patchy infiltrate (18 cases, 81.8%) and bronchiectasis (8 cases, 36.4%). Lesions with high FDG-uptake (SUV_I−Lung_ > 2.5) were observed in 19 patients (Fig. 2A, blue arrows) and those with low FDG-uptake (SUV_I−Lung_ ≤ 2.5) were observed in 3 patients (Fig. 2B, white arrows).

#### Extra-pulmonary lesions

Extra-pulmonary lesions with abnormally high ^18^F-FDG FDG uptake were found in 17 patients (73.8%). Among them, ^18^F-FDG PET/CT showed increased ^18^F-FDG accumulation in the lymph node (14 cases), bone (6 cases), subcutaneous tissue (1 case), the spleen (1 case), and adrenal gland (1 case). Lesions with high FDG-uptake (SUV_E−Lung_ > 2.5) were observed in 15 patients, and those with low FDG-uptake (SUV_E−Lung_ ≤ 2.5) were observed in 2 patients.

#### Extent of lesion involvement

**Fig. 3:**
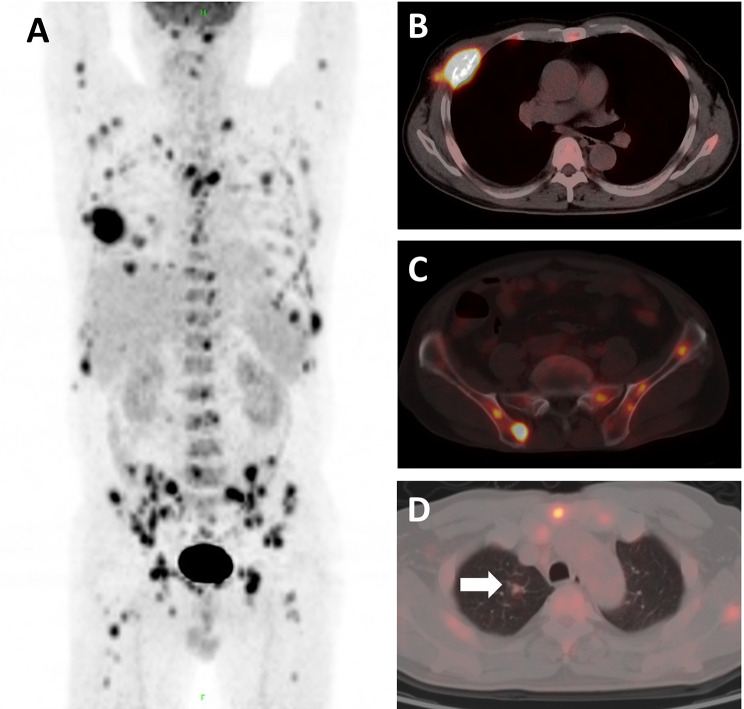
A 47-year-old man (No.2) presented with right calf and chest pain, fever, and sweating for one month. Laboratory examination revealed significant increases in leukocyte count (19 × 10^9^/L), erythrocyte sedimentation rate (77 mm/hour), and C-reactive protein level (87 mg/L). ^18^F-FDG PET/CT showed diffuse bony destruction with increased high FDG uptake (SUV_max_ = 14.8) and mild-uptake small nodule in the upper lobe of the right lung (white arrows, SUV_max_ = 2.0). Finally, *M. intracellulare* was confirmed by sputum culture of a rib lesion after removal in orthopedics

Disseminated NTM infection was observed in 17 patients using ^18^F-FDG PET/CT. Among them, involvements of the common multi-system were found in the lung and lymph nodes (9 cases), and lung and bone (3 cases) (Fig. 3). Other multi-system involvements included lung, lymph node, bone and subcutaneous tissue (1 case), lung, lymph node and bone (1 case), lung, lymph node and spleen (1 case), lung, lymph node and adrenal gland (1 case), and lymph node and bone (1 case). Localized pulmonary NTM infection was identified in 6 patients.

### Metabolic parameters

The median SUV_Top_ in 23 patients with lung involvement was 8.2 ± 5.7 (range: 1.2–28.1); the median SUV_I−Lung_ in 22 patients with lung involvement was 6.6 ± 3.9 (range: 1.2–13.9); the median SUV_E−Lung_ in 17 patients was 6.3 ± 6.3 (range: 1.8–28.1); the median SUV_Liver_, SUV_Marrow_ and SUV_Spleen_ were 2.7 ± 0.6 (range: 1.7–3.8), 3.7 ± 2.0 (range: 1.7–6.7), and 2.5 ± 0.9 (range: 1.6-6.0), respectively; the median SUR_Liver_ and SUR_Blood_ were 3.7 ± 2.3 (range: 0.5–9.1) and 5.9 ± 3.9 (range: 0.6–16.4), respectively; the median MLV and TLG were 122.2 ± 211.7 (range: 0-972.6) and 526.9 ± 992.1 (range: 0-4628.6), respectively.

### ^18^F-FDG PET/CT characteristics in different immune states, extent of lesion involvement, and severity of NTM-PD

**Fig. 4:**
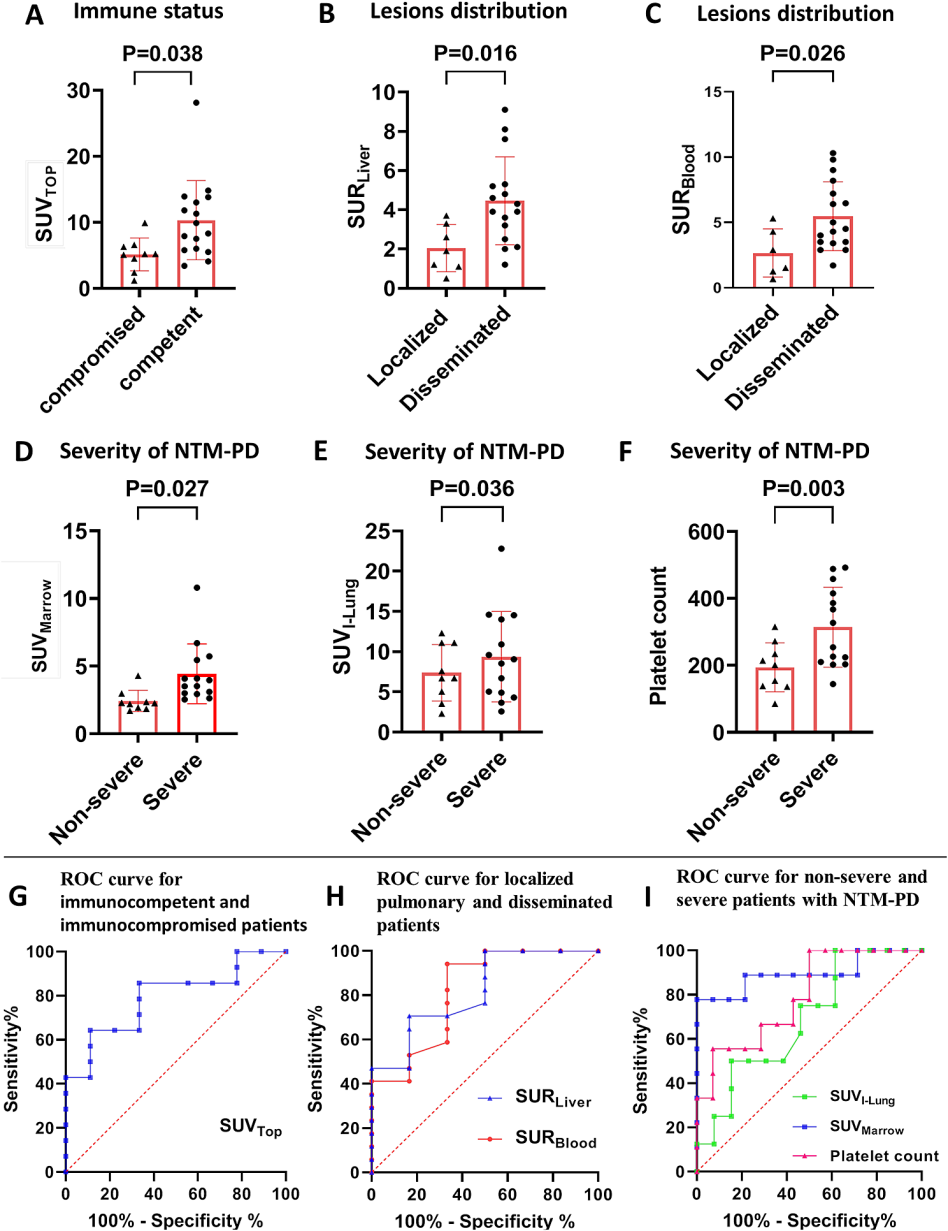
(**A**). Comparison of SUV_Top_ between immunocompromised and immunocompetent patients. (**B**). Comparison of SUR_Liver_ between the localized pulmonary diseases group and the disseminated diseases group. (**C**). Comparison of SUR_Blood_ between the localized pulmonary diseases group and the disseminated diseases group. (**D**). Comparison of SUV_Marrow_ between severe NTM-PD and non-severe NTM-PD. (**E**). Comparison of SUV_I−Lung_ between severe NTM-PD and non-severe NTM-PD. (**F**). Comparison of platelet count between severe NTM-PD and non-severe NTM-PD. (**G**). ROC curve analysis for predicting immunocompromised status in NTM patients using SUV_Top_ values of ^18^F-FDG-PET/CT scans. (**H**). ROC curve analysis for predicting disseminated NTM infection using SUR_Liver_ and SUR_Blood_ values of ^18^F-FDG-PET/CT scans. (**I**). Receiver operating characteristic curve (ROC) analysis for predicting severe NTM-PD using platelet count, and SUV_Marrow,_ and SUV_I−Lung_ values of ^18^F-FDG-PET/CT scans

The comparison of clinical and ^18^FDG PET/CT characteristics in patients with different immune states, extent of lesion involvement, and severity of NTM-PD were shown in Table 3; Fig. 4. As for the immune state, SUV_Top_ in immunocompetent patients was significantly higher than that in immunocompromised patients (median SUV_Top_, 10.0 ± 6.4 vs. 5.2 ± 2.5, *P* = 0.038, Fig. 4A). As for extent of lesion involvement, SUR_Liver_ and SUR_Blood_ in disseminated disease were significantly higher than those in localized pulmonary disease (median SUR_Liver_, 3.8 ± 1.6 vs. 1.9 ± 1.1, *P* = 0.016, Fig. 4B; median SUR_Blood_, 5.5 ± 2.6 vs. 2.7 ± 1.8, *P* = 0.026, Fig. 4C). As for the severity of NTM-PD, the median SUV_Marrow_, and SUV_I−Lung_ in patients with severe NTM-PD were significantly higher than those in patients with non-severe NTM-PD (median SUV_Marrow_, 4.4 ± 2.0 vs. 2.4 ± 0.8, *P* = 0.027, Fig. 4D; median SUV_I−Lung_, 7.7 ± 4.3 vs. 4.3 ± 2.3, *P* = 0.036). There were no significant differences in the metabolic parameters of MLV and TLG between the localized pulmonary disease group and the disseminated disease group, the severe and non-severe NTM-PD group, and the immunocompetent and immunocompromised patients.

**Table 3:** Comparison of clinical and ^18^FDG PET/CT characteristics among groups with different immune status, extent of lesion involvement and severity of NTM-PD

Characteristics	Immune status			Extent of lesion involvement			Severity of NTM-PD		
	Immunocompromised	Immunocompetent	P	Localized	Disseminated	P	Severe	Non-severe	P
White cell count(× 10^9^/L )	6.2 ± 4.3	10.1 ± 4.8	0.062	8.4 ± 4.1	8.6 ± 5.3	0.882	9.4 ± 5.6	7.4 ± 3.5	0.354
Neutrophilic granulocyte count (× 10^9^/L )	4.2 ± 3.8	7.9 ± 4.9	0.068	5.7 ± 4.1	6.8 ± 5.1	0.642	7.2 ± 5.6	5.3 ± 2.8	0.378
lymphocyte count(× 10^9^/L )	1.3 ± 1.2	1.4 ± 0.9	0.807	1.5 ± 1.4	1.3 ± 0.9	0.620	1.5 ± 1.2	1.1 ± 0.5	0.357
Red blood cell count(× 10^9^/L )	3.5 ± 1.0	3.9 ± 0.7	0.184	3.9 ± 0.8	3.7 ± 0.8	0.685	3.7 ± 1.0	3.8 ± 0.5	0.945
Hemoglobin (g/dl)	102.7 ± 32.3	112.7 ± 22.7	0.386	117.7 ± 25.2	104.9 ± 7.1	0.301	107.0 ± 30.4	111.7 ± 20.9	0.692
Platelet count (× 10^9^/L )	240.0 ± 104.7	284.7 ± 126.3	0.386	225.4 ± 115.6	285.4 ± 117.9	0.272	314.0 ± 119.3	194.3 ± 73.1	0.003*
C-reactive protein (mg/L)	37.2 ± 36.1	64.3 ± 64.8	0.271	19.0 ± 10.1	64.9 ± 60.4	0.066	56.3 ± 55.1	42.5 ± 56.7	0.569
Erythrocyte sedimentation rate (mm/H)	46.7 ± 33.6	43.5 ± 35.8	0.879	27.6 ± 15.3	36.6 ± 34.9	0.220	40.7 ± 38.7	10.6 ± 16.2	0.040
The percentage of total T lymphocyte (%)	766.0 ± 726.7	1032.2 ± 444.2	0.494	60.9 ± 28.6	63.8 ± 27.9	0.880	69.6 ± 23.3	51.6 ± 31.8	0.305
IL-6 (pg/ml)	35.6 ± 57.5	23.6 ± 44.7	0.579	36.3 ± 65.8	24.7 ± 42.1	0.615	39.9 ± 60.8	10.2 ± 7.1	0.163
TNF-α (pg/ml)	11.8 ± 24.2	3.0 ± 2.1	0.127	10.6 ± 2.6	21.8 ± 2.1	0.221	21.1 ± 2.5	9.4 ± 3.1	0.341
Lactate dehydrogenase (U/L)	218.8 ± 52.6	196.4 ± 66.3	0.404	214.9 ± 55.9	200.0 ± 64.5	0.626	217.6 ± 68.4	185.9 ± 44.3	0.233
SUV_I−lung_	5.5 ± 3.9	7.2 ± 3.9	0.338	5.15 ± 3.55	6.7 ± 4.3	0.437	7.7 ± 4.3	4.3 ± 2.7	0.036*
SUV_E−Lung_	5.2 ± 2.8	6.9 ± 7.5	0.606	NA^#^	NA	NA	8.5 ± 7.4	4.5 ± 2.1	0.214
SUV_Top_	5.2 ± 2.5	10.0 ± 6.4	0.038*	5.6 ± 3.6	9.3 ± 6.1	0.153	9.0 ± 3.7	7.5 ± 7.1	0.559
SUR_Liver_	2.7 ± 1.4	4.3 ± 0.2.5	0.095	1.9 ± 1.1	3.8 ± 1.6	0.016*	3.4 ± 1.2	2.8 ± 2.3	0.464
SUR_Blood_	4.7 ± 2.9	6.6 ± 4.3	0.258	2.7 ± 1.8	5.5 ± 2.6	0.026*	5.3 ± 2.1	4.3 ± 3.3	0.410
SUV_Liver_	2.6 ± 0.6	2.7 ± 0.7	0.825	2.7 ± 0.4	2.6 ± 0.7	0.762	2.7 ± 0.6	2.6 ± 0.6	0.586
SUV_Marrow_	3.6 ± 1.6	3.7 ± 2.3	0.896	3.0 ± 0.08	3.9 ± 2.3	0.320	4.4 ± 2.0	2.4 ± 0.8	0.027*
SUV_Spleen_	2.7 ± 1.4	2.4 ± 0.4	0.444	2.0 ± 1.0	2.4 ± 1.3	0.489	2.5 ± 1.3	1.8 ± 0.7	0.131
MLV(cm^3^)	56.4 ± 50.2	172.6 ± 261.9	0.206	35.6 ± 59.0	160.1 ± 245.0	0.204	116.2 ± 135.9	127.7 ± 271.5	0.900
TLG(ml*SUV)	237.1 ± 256.8	748.3 ± 1230.9	0.236	119.7 ± 210.9	705.1 ± 1149.9	0.201	465.9 ± 559.1	582.9 ± 1299.1	0.785

^18^F-FDG PET/CT: ^18^F-fluorodeoxyglucose positron emission tomography/computed tomography; NTM: Non-tuberculous mycobacterial infection; NTM-PD: Non-tuberculous mycobacterial pulmonary diseases; SUV_Top_: The most FDG-avid lesion in the whole body; SUV_I−lung_: SUV_max_ of lung lesions, SUV_E−lung_: SUV_max_ of extra-lung lesions; SUV_Liver_: SUV_max_ of liver; SUV_Spleen_: SUV_max_ of spleen; SUV_Marrow_: SUV_max_ of marrow; SUR_Liver_: SUV_max_ ratio of lesion-to-liver; SUR_Blood_: SUV_max_ ratio of lesion-to-blood pool. MLV: the metabolic lesion volume; TLG: total lesion glycolysis

NA^#^: Localized groups were defined as NTM patients with only pulmonary involvement

*: *P* value less than 0.05 was considered statistically significant

Receiver operating characteristic (ROC) curves were drawn to evaluate the consistency between the ^18^F-FDG PET/CT metabolic parameters and clinical features (Fig. 4). The area under the curve (AUC) values in immunocompromised and immunocompetent groups were 0.794 for SUV_Top_ (*P* = 0.02) (Fig. 4G). The cut-off value of SUV_Top_ was 7.1, and the sensitivity and specificity of ^18^F-FDG PET/CT for immunocompromised states in NTM patients were 88.9% and 64.3%, respectively. The AUC in group of the localized pulmonary NTM infection and the disseminated NTM infection group were 0.819 for SUR_Liver_ (*P* = 0.023) and 0.814 for SUR_Blood_ (*P* = 0.025) (Fig. 4I). When the cut-off values of SUR_Liver_ and SUR_Blood_ were 2.8 and 4.4, respectively, the sensitivity and specificity of ^18^F-FDG PET/CT for disseminated NTM infection were 83.3% and 70.6%, and 83.3% and 52.9%, respectively. The AUC in the severe and non-severe groups were 0.897 for SUV_Marrow_ (*P* = 0.001) and 0.688 for SUV_I−Lung_ (*P* = 0.158), respectively (Fig. 4H). When the cut-off values of SUV_Marrow_ and SUV_I−Lung_ were 3.1 and 4.6, respectively, the sensitivity and specificity of ^18^F-FDG PET/CT for the identification severity of NTM-PD were 76.9% and 88.9%, and 76.9% and 55.9%, respectively.

### Correlations between the metabolic parameters of ^18^F-FDG PET/CT and the clinical laboratory results

**Fig. 5:**
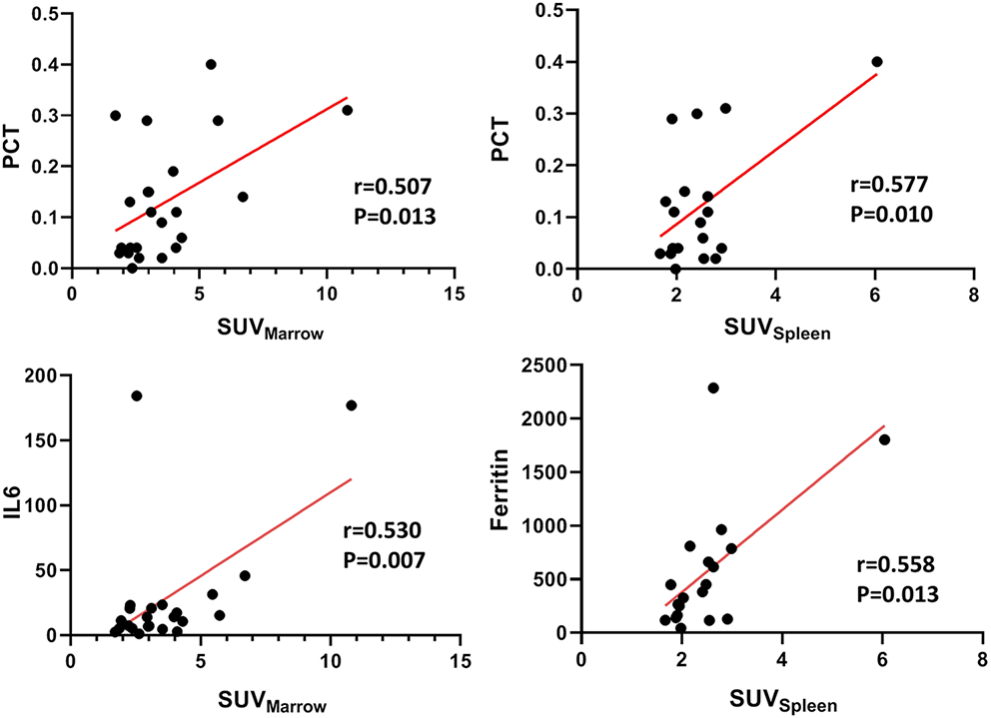
Spearman’s rank analysis of ^18^F-FDG uptake (SUV_Spleen_ and SUV_Marrow_) and laboratory markers such as interleukin 6 (IL-6), procalcitonin (PCT) and ferritin

Spearman’s correlation analysis (Fig. 5) revealed a significant positive correlation between serum PCT, IL-6 and SUV_Marrow_ (*r* = 0.507 and 0.530, *p* < 0.05). Furthermore, serum PCT and ferritin were significantly positively related to SUV_Spleen_ (*r* = 0.577 and 0.558, respectively, *p* < 0.05).

## Discussion

Non-tuberculous mycobacteria (NTM) are ubiquitously present in the environment and include opportunistic pathogens that can cause various diseases in multiple organs in both immunocompromised and immunocompetent individuals. Recently, the prevalence of NTM diseases have increased globally, which are now recognized to represent an emerging public health problem [1]. According to established criteria for NTM disease treatment [10, 11], it strongly recommended that individuals receive a period of longitudinal assessment (including immune status, the severity and risk of progressive NTM infection, and the extent of lesion involvement) before a decision is made to start treatment. Therefore, it is critical to develop noninvasive imaging tools for evaluating active lesions, immune status, and the extent of lesion involvement [12]. Previous studies have revealed the usefulness of ^18^F-FDG PET in differentiating latent from active TB and evaluating early therapeutic response to anti-TB. Moreover, high FDG-avid lesions in a patient with TB may also reflect a host immune system response that will ultimately prevail [26].

^18^F-FDG PET/CT has revolutionized oncology care owing to its mechanism of action, including being taken up by cell surface transporters (mainly the glucose transporter-1 and 3, GLUT-1 and 3) and being transformed by the rate-limiting glycolytic enzyme hexokinase into FDG-6-phosphate [13]. Infection and inflammation contribute to enhancing vascular permeability and the release of inflammatory mediator expressing increased levels of GLUT-1 and 3 (macrophages, neutrophils), as well as increased HK activity, resulting in greater ^18^F-FDG delivery to affected sites [13]. Furthermore, ^18^F-FDG PET/CT has been used for diagnosing tumor, infection, fever of unknown origin and assessing therapy response due to a whole-body imaging and changes in FDG metabolic parameters ahead of morphological changes by conventional imaging techniques (CT and MRI). Therefore, ^18^F-FDG PET/CT has been widely used not only for the diagnosis of infectious and inflammatory diseases but also in the evaluation active stage of systemic disease [13]. SUV_max_ and SUR are the most commonly used imaging biomarkers to assess FDG uptake levels. Besson FL et al. [27] indicated that there was a significant improvement in specificity by using SUR_Liver_ > 1 compared to visual analysis. In the present study, we collected ^18^F-FDG PET/CT scanning data from 22 patients with NTM-PD with a median SUV_max_ of 6.6 (range: 1.2–13.9), which was higher than that reported previously (median SUV_max_ of 4.2) [28]. It may possible be more patients with disseminated NTM (17/23) in our study. Moreover, ^18^F-FDG PET/CT showed an intense or moderate ^18^F-FDG uptake in both intra-pulmonary and extra-pulmonary lesions in 21 patients, and high FDG-uptake (SUV_max_ > 2.5) was observed in 19 patients with intra-pulmonary lesions and 15 patients with extra-pulmonary lesions, suggesting an active stage of the disease. Sinner V et al. [29] reported that the activity of the chorioretinal lesions with NTM infection corresponded to disease progression. The above evidences highlight that ^18^F-FDG PET/CT can be of great value to assess the extent of active NTM lesion, particularly for extra-pulmonary lesions, since obtaining tissue or fluid for culture or mNGS may be not always possible or too invasive.

NTM are opportunistic pathogens to humans. Exposure to NTM organisms in the environment in common in day-to-day life, while NTM diseases occur infrequently due to their lower virulence than *Mycobacterium tuberculosis.* However, to date, it has become apparent that some individuals are prone to NTM diseases, particularly immunosuppressed patients with reduced cell-mediated immunity [3–5]. The rate of glucose utilization is accentuated by immune cell activation during inflammation and infection. ^18^F-FDG PET/CT has provided the most robust evidence regarding this phenomenon in the initial and treatment response assessment of IFD (invasive fungal disease) in immunocompromised patients [30]. The cases selected for our study included 9 (39.1%) immunosuppressed patients and 14 (60.9%) immunocompetent patients. SUV_Top_ in immunocompetent patients was significantly higher than that in immunocompromised patients. Moreover, the cut-off value of SUV_Top_ was 7.1, and the sensitivity and specificity of ^18^F-FDG PET/CT for compromised immune status in NTM patients were 88.9% and 64.3%, respectively. These results indicated that SUV_Top_ was significantly positively related to immune cell activation due to the upregulation of GLUT-1 and 3, along with increased hexokinase activity [13], whereas immunosuppressed patients exhibit the disruption or depletion of cell-mediated immunity and immune cell activation in immunocompromised patients is lower than that in immunocompetent patients. NTM guidelines [10, 11] strongly recommend that immunocompromised patients with NTM infection require a longer duration and dose of treatment. In future work, we aim to expand the sample size and conduct prospective experiments to further explore the relationship of immune function and metabolic parameters of FDG PET/CT in NTM patients.

NTM can affect any organ in the body, and the main clinical manifestations in adults are localized NTM pulmonary disease and/or other disseminated diseases. Disseminated NTM diseases (defined as the involvement of two or more non-contiguous body organs) often occur in patients with an immune defect, and 2–8 per cent of patients with disseminated diseases may have concurrent pulmonary involvement [5]. The *Mycobacterium avium* complex (MAC) and rapidly growing mycobacteria (RGM) have been identified as important pathogens contributing disseminated diseases [31]. Thus, early diagnosis and treatment is essential to minimize morbidity and costs, as well as prevent long-term disability [5]. In this study, extra-pulmonary lesions with abnormally high ^18^F-FDG FDG uptake were found in 17 patients (73.8%) with disseminated diseases. In these patients, ^18^F-FDG PET/CT showed increased ^18^F-FDG accumulation in the lymph node (14 cases), bone (6 cases), subcutaneous tissue (1 case), spleen (1 case), and adrenal gland (1 case). The metabolic parameters SUR_Liver_ and SUR_Blood_ in disseminated diseases were significantly higher than those in localized pulmonary diseases. When the cut-off values of SUR_Liver_ and SUR_Blood_ were 2.8 and 4.4, respectively, the sensitivity and specificity of ^18^F-FDG PET/CT for disseminated diseases were 83.3% and 70.6%, and 83.3% and 52.9%, respectively. These results showed that an active NTM lesion readily takes up ^18^F-FDG, both in pulmonary and extra-pulmonary lesions. Moreover, NTM patients with high SUR_Liver_ and SUR_Blood_ level were related to disseminated diseases. This result needs to be confirmed in the next prospective study.

According to the guidelines for the management of non-tuberculosis mycobacterial pulmonary disease by the British Thoracic Society [11], as the treatment of NTM pulmonary disease is arduous, lengthy and costly, several factors should be considered before a decision is made to begin treatment, such as the severity of NTM-PD, the risk of progressive NTM-PD, the presence of any comorbidities, and the goals of treatment [9, 12]. ^18^F-FDG PET/CT is a functional imaging technique that detects metabolically active areas by measuring the accumulation of ^18^F-FDG, and may help to establish NTM-PD diagnoses [32]. In the present study, when the cut-off values of SUV_Marrow,_ and SUV_I−lung_ were 3.1 and 4.6, respectively, the sensitivity and specificity of ^18^F-FDG PET/CT for the identification of NTM-PD severity were 76.9% and 88.9%, and 76.9% and 55.9%, respectively. A high FDG uptake of NTM-PD lesion indicates inflammatory activity, significant mycobacterial pathogenicity, or an elicited systemic response (such as in Patient 1 in Fig. 2), while normal or low FDG uptake shows inflammation regression or scar formation (such as in Patient 2 in Fig. 2). Therefore, the indices SUV_I−Lung_ and SUV_Marrow_ can both suggest systemic inflammatory response, either directly after NTM infection or indirectly by reflecting the virulence of the bacteria. NTM-PD patients with SUV_Marrow_ ≥ 3.1 and SUV_I−Lung_ ≥ 4.6 may likely be severe NTM if effective treatment is delayed. Therefore, these metabolic parameters can contribute to the planning of appropriate treatment for NTM. Furthermore, the Spearman’s correlation analysis revealed a significant positive correlation between serum IL-6 and SUV_Marrow_, ferritin and SUV_Spleen_. The PET metabolic parameters of bone marrow and spleen may reflect the development and progression of a cytokine storm [33]. To this end, the value of ^18^F-FDG PET/CT in predicting immune cell activation and evaluating treatment response of NTM patients deserves further investigation.

This study inevitably has some limitations. First, it was conducted at a single center, and the sample size was limited (*n* = 23). All images were acquired on the same PET/CT scanner model (Biograph version; Siemens, Germany), imaging protocol, and reconstruction. Second, because this was a retrospective study, the use of ^18^F-FDG PET/CT in the assessment of patients potentially introduced a bias toward a disseminated NTM infection cohort. Third, in our study, each patient underwent an ^18^F-FDG PET/CT scan only once (baseline assessment in 16 patients and anti-NTM pre-PET in 7 patients) and lacked post-treatment reassessment. Fourth, 10 patients were misdiagnosed malignancies (multiple myeloma, lung cancer and lymphadenoma, etc.) by ^18^F-FDG PET/CT (Table 1). So, ^18^F-FDG PET/CT has a limited role in differentiating disseminated NTM diseases from malignancies. Lastly, metabolic measurements reflected the FDG uptake by inflammatory cells rather than NTM. Thus, NTM-specific radiolabeled compounds and imaging approaches urgently need to be developed. In addition, a multicenter, prospective analysis should be designed in a future study to attain a better representation of NTM-infected patients.

## Conclusion

^18^F-FDG PET/CT is a useful tool in the diagnosis, evaluation of disease activity, immune status, and extent of lesion involvement in NTM patients, and can contribute to planning the appropriate treatment for NTM.

### Electronic supplementary material

Below is the link to the electronic supplementary material.

Supplementary Material 1

## Abbreviations

NTM: Non-tuberculous mycobacteria
NTM-PD: Non-tuberculous mycobacterial pulmonary diseases
MDS: Myelodysplastic syndromes
AIDS: Acquired immunodeficiency syndrome
COPD: Chronic obstructive pulmonary disease
BALF: Bronchoalveolar lavage fluid
NTM-DNA-PCR: PCR assay for non-tuberculous mycobacteria DNA
IIP: Idiopathic interstitial pneumonia
mNGS: Metagenomic next-generation sequencing
^18^F-FDG PET/CT: ^18^F-fluorodeoxyglucose positron emission tomography/computed tomography
CT: Computed tomography
MRI: Magnetic resonance imaging
SUV_Top_: The most FDG-avid lesion in the whole body
SUV_I−Lung_: SUV_max_ of intra-pulmonary lesions
SUV_E−Lung_: SUV_max_ of extra-pulmonary lesions
SUV_Liver_: SUV_max_ of the liver
SUV_Spleen_: SUV_max_ of the spleen
SUV_Marrow_: SUV_max_ of the marrow
SUR_Liver_: SUV_Top_/SUV_max_ of the liver (SUR_Liver_)
SUR_Blood_: SUV_Top_/SUV_max_ of the blood (SUR_Blood_)
MLV: Metabolic lesion volume
TLG: Total lesion glycolysis
ROC: Receiver operating characteristic curve
AUCs: Areas under the curve
