# Supplementary material for: The additional value of 18F-FDG PET/CT imaging in guiding the treatment strategy of non-tuberculous mycobacterial patients
Source: Respir Res. 2024 Mar 18;25:132. doi: 10.1186/s12931-024-02757-7 (PMC10949717; doi:10.1186/s12931-024-02757-7)
Supplement: Supplementary file 1 — Supplementary Material 1 [file 12931_2024_2757_MOESM1_ESM.docx]

| **T****able 2** **Clinical laboratory results** | | | |
| --- | --- | --- | --- |
| Laboratory examination  White cell count(× 10^9^/L)  Absolute neutrophil count (× 10^9^/L)  Absolute lymphocyte count (× 10^9^/L)  Platelet count (× 10^9^/L)  Red cell count(× 10^9^/L)  Hemoglobin (g/dl)  C-reactive protein (mg/L)  Erythrocyte sedimentation rate (mm/H)  Procalcitonin (μg/L)  Lactate dehydrogenase (U/L)  Ferritin(ng/ml)  CA125 (U/L)  CA199 (U/L)  Creatinine (μmol/L)  T-spot, nagetive, (n, %)  Serum cytokines  IL-2 (pg/ml)  IL-4 (pg/ml)  IL-6 (pg/ml)  IL-10 (pg/ml)  TNF-α (pg/ml)  IFN-γ (pg/ml)  Cellular immune function in peripheral blood (%)  Total T lymphocyte (%)  Assisted/induced T lymphocyte (%)  Inhibited/cytotoxic T lymphocyte (%)  B lymphocyte (%)  NK cell (%) | median (range)  7.6 (2.3-22.8)  5.1 (1.6-20.2)  1.0 (0.3-4.5)  226(85-492)  3.9 (2.1-5.1)  110 (56-154)  35.6 (0.11-176)  30 (5-119)   - 1. (0.002-0.4)   197.5(105-329)  326.6(43.2-2286.6)  34.6(2.1-290.8)  6.4(2.0-186.8)  69(52-175)  19(82.6)  0.6(0.1-2.0)  0.8(0.10-4.7)  13.9 (1.1-184.3)  2.0(0.1-8.2)  3.5(0.1-75.4)  2.4(0.1-26.4)  62.1(18.7-96.1)  26.8(5.4-53.3)  26.1(6.0-77.7)  8.9(0.3-20.2)  10.3(3.2-32.5) | Reference range  4.0-10.0  2.0-7.0  0.8-4.0  83-303  4.09-5.74  131-172  0.00-8.00  0-15  0-0.5  109-245  21.8-274.7  0-35  0-37  59-104  /  0.10-4.10  0.10-3.20  0.10-2.90  0.10-5.00  0.10-23.00  0.10-18.00  50.00-87.00  21.00-51.00  12.00-47.00  3.00-19.00  3.00-37.00 | Above or below the reference range (n, %)  10(43.5)↑  9(39.1)↑  1(4.3)↑  8(34.8)↑  8(34.8)↓  11(47.8)↓  16(69.6)↑  15(65.2)↑  0(0)↑  3(13.0)↑  13(56.5)↑  9(39.1)↑  3(13.0)↑  0(0)↑  /  0(0)↑  0(0)↑  20(87.0)↑  1(4.3)↑  2(8.7)↑  2(8.7)↑  2(8.7)↑  2(8.7)↓  2(8.7)↓  1(8.7)↓  0(0)↓ |

↑: Above the reference range; ↓:Below the reference range
